# Biceps breakdown: a case of spontaneous necrotizing myositis

**DOI:** 10.1093/jscr/rjaf287

**Published:** 2025-05-07

**Authors:** Jai Pantling, Theodore Howard, Fionnuala O’Leary

**Affiliations:** The Old Schools, University of Cambridge, Trinity Lane, Cambridge CB2 1TN, United Kingdom; Department of Plastic Surgery, Cambridge University Hospitals, Hills Road, Cambridge CB2 0QQ, United Kingdom; Department of Plastic Surgery, Cambridge University Hospitals, Hills Road, Cambridge CB2 0QQ, United Kingdom

**Keywords:** necrotizing myositis, necrotizing soft tissue infection, spontaneous, biceps brachii

## Abstract

We report a case of spontaneous necrotizing myositis of the biceps brachii in an immunocompetent patient with no comorbidities. We highlight the diagnostic challenges with this case, the utility of computed tomography in diagnosing soft tissue infections, and the importance of source control in septic patients.

## Introduction

Necrotizing soft tissue infections (NSTIs) are rare but life-threatening conditions with a mortality rate of 36.5% [1]. The most common presentation is necrotizing fasciitis, where bacteria spread along the superficial fascial planes, clinically manifesting as rapidly progressive painful cellulitis and skin necrosis, which typically progresses from sepsis to multi-organ failure [2]. We describe a rare case of necrotizing myositis, which presented in a previously healthy and immunocompetent patient, without the typical features of necrotizing fasciitis, and highlight the diagnostic and clinical challenges in managing this potentially fatal condition.

## Case

**Figure 1:**
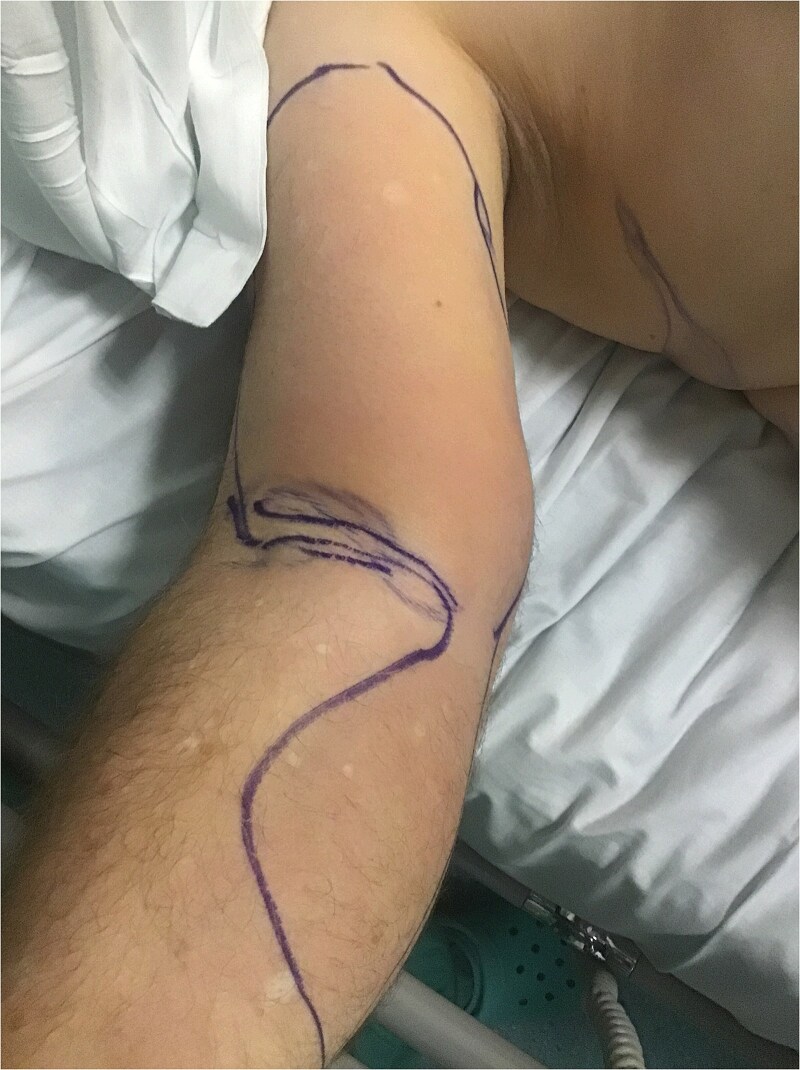
Right arm at presentation.

A 68-year-old previously fit and well male presented to our accident and emergency department with a 2-day history of malaise, arm swelling, and neck stiffness. He was haemodynamically unstable. Clinical examination showed severe pain in the anterior arm with loss of active flexion. Aside from mild erythema, there was no evidence of epidermolysis, skin necrosis or crepitus in keeping with typical necrotizing fasciitis (Fig. 1).

A full blood count and renal profile showed neutrophilia, lymphopaenia, and uraemia, with a significantly raised serum creatinine and C-reactive protein. Blood cultures grew gram-positive cocci and contained *Staphylococcus aureus* toxin. An ultrasound Doppler and computed tomography (CT) scan of the head were ordered due to a significantly raised D-dimer. Both were negative and ruled out upper limb or cerebral thrombus formation.

**Figure 2:**
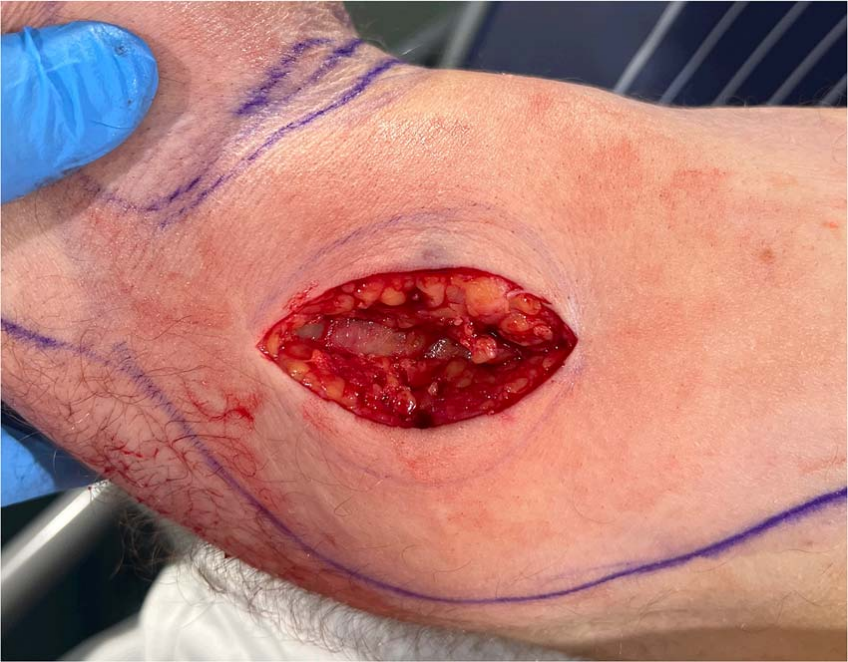
Negative sweep test.

**Figure 3:**
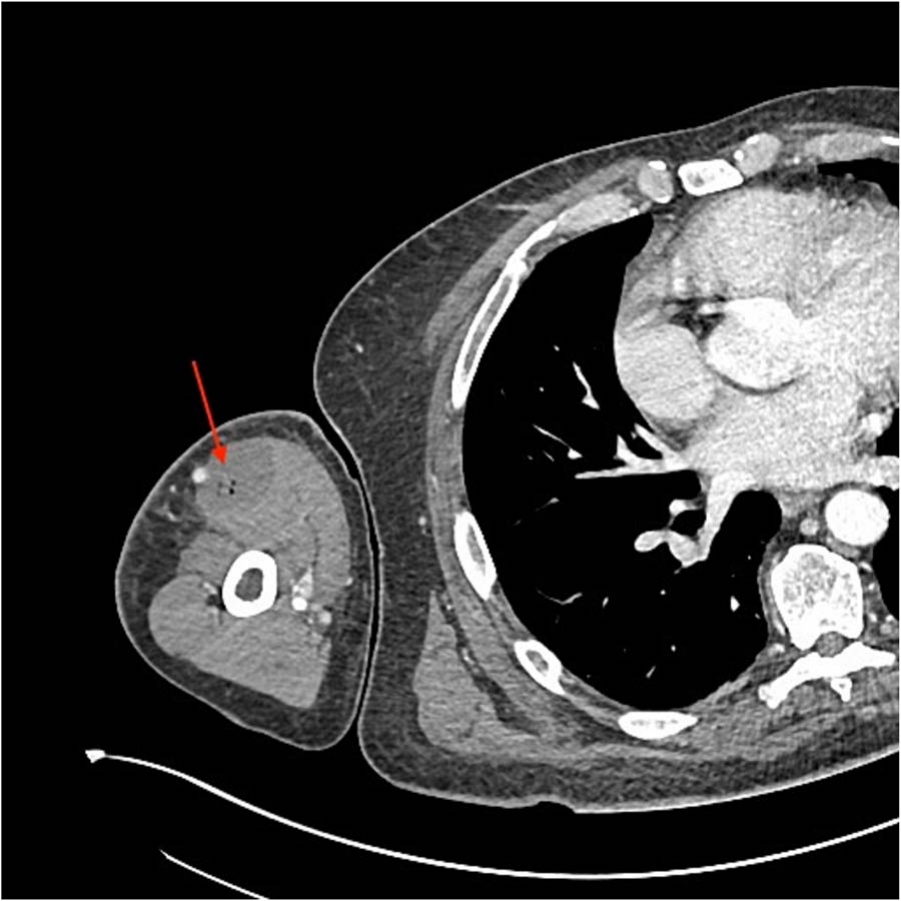
CT scan showing gas loculi within the biceps (arrow).

An urgent review by the plastic surgery team prompted a bedside sweep test to assess for necrotizing fasciitis. The dermis, fat and fascia did not show any evidence of venous thrombosis or typical ‘dishwater fluid’, synonymous with typical necrotizing fasciitis (Fig. 2). This is a negative test for necrotizing fasciitis (negative predictive value = 100%) [3]. With no clear cause of the sepsis and progressive limb swelling, a CT scan of the arm was requested. The report described a fluid collection containing gas locules within the biceps (Fig. 3).

**Figure 4:**
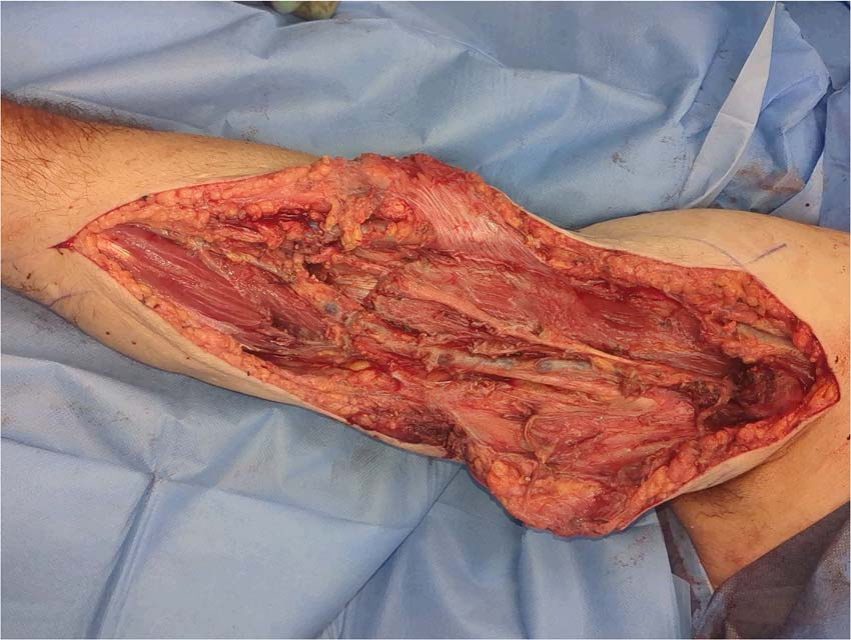
Right arm after debridement.

The patient was urgently brought to theatre for fasciotomy and debridement under general anaesthesia. Deep to the healthy fat and fascia, the biceps brachii was found to be unreactive and unhealthy. Pus was released from the intermuscular septum of the biceps. Samples were sent for culture and sensitivity. The biceps was resected, and debridement continued to healthy, reactive, and bleeding muscle (Fig. 4). The patient was transferred to intensive care following surgery.

A second washout was performed at 36 h due to ongoing bacteraemia and continued swelling of the shoulder despite presumed infection source control. The initial wounds were extended proximally however no further necrotic muscle was encountered.

Infective endocarditis is a typical cause of infective myositis, however, both transthoracic echocardiogram on day 2 and transoesophageal echocardiogram on day 5 did not demonstrate any compelling evidence of this.

As the patient remained septic with ongoing raised inflammatory markers, a second opinion was sought from an MSK radiologist of the original upper arm CT. The updated report confirmed the presence of previously undiagnosed collections deep to the deltoid, involving infraspinatus and subscapularis, which necessitated an additional return to theatre for washout of the posterior shoulder. Entry was obtained between infraspinatus and teres major, and a large volume of pus was expressed. On day 9, the patient underwent a fourth washout of the arm and a second washout of the shoulder, with necrotic portions of the latissimus dorsi being debrided. On day 29, a final debridement and washout were performed due to rising inflammatory markers and temperatures. Haemo-serous fluid was drained, but not pus.

Samples continued to grow *S. aureus* and Cutibacterium acne. On day 29, *Klebsiella pneumoniae* was cultured. Resultingly, the patient had numerous courses of antibiotics. He spent 19 days in the intensive care unit and was eventually discharged on day 69. He will be followed up in the outpatient-parenteral antimicrobial therapy clinic and has regained elbow flexion such that further reconstructive surgery is not required.

## Discussion

Due to the initial presentation of limb weakness and sepsis of unknown origin, this case represented a diagnostic challenge with differentials of upper limb venous thrombosis, cerebrovascular event, and necrotizing fasciitis excluded before cross-sectional imaging confirmed necrotizing myositis. Despite our patient having a Laboratory Risk Indicator for Necrotizing Fasciitis (LRINEC) score of 10, with scores greater than 6 having a positive predictive value of 92% [4], a sweep test demonstrated that an alternative diagnosis was more likely. Hence, a CT was performed due to further deterioration and lack of any other plausible diagnosis. It found evidence of an NSTI in the biceps. CT has a sensitivity of 88.5% in diagnosing NSTIs [5]. With no obvious cause preceding the soft tissue infection and a lack of any risk factors, a transthoracic echocardiogram was ordered. However, this did not find any evidence of any infective endocarditis. It was highly unusual to find no evidence of a deep source of infection. So, spontaneous necrotizing myositis became the working diagnosis.

Other cases that have resulted in myositis appear to have an earlier pre-disposing infection with Group A Streptococcus. For example, one case describes a 22-year-old woman who was also suffering from cavitating pneumonia [6], whilst another presents a 27-year-old patient who had myositis preceded by a 10-day pharyngeal infection [7]. Conversely, our patient reported 5 days of pain and stiffness in the neck and 2 days of tenderness and swelling in the arm, both of which can be attributed to necrotizing myositis itself.

In our patient, the initial CT report described a fluid collection within the biceps muscle with gas locules, suspicious of necrotizing fasciitis. This guided the immediate surgical management and hence debridement of the infected muscle. However, because the source of infection was not adequately controlled, the patient continued to deteriorate. Only when the CT was re-reported by an MSK radiologist that collections deep to the deltoid and involving infraspinatus and subscapularis were noted and could subsequently debrided. This highlights the clinical and radiological diagnostic challenges with this case.
